# Active elite rugby participation is associated with altered precentral cortical thickness

**DOI:** 10.1093/braincomms/fcad257

**Published:** 2023-10-06

**Authors:** Thomas D Parker, Karl A Zimmerman, Etienne Laverse, Niall J Bourke, Neil S N Graham, Emma-Jane Mallas, Amanda Heslegrave, Henrik Zetterberg, Simon Kemp, Huw R Morris, David J Sharp

**Affiliations:** 1 Department of Brain Sciences, Imperial College London, London, W12 0BZ, UK; Dementia Research Institute Care, Research and Technology Centre, Imperial College London, London, W12 0BZ, UK; UCL Institute of Neurology, London, WC1N 3BG, UK; 1 Department of Brain Sciences, Imperial College London, London, W12 0BZ, UK; Dementia Research Institute Care, Research and Technology Centre, Imperial College London, London, W12 0BZ, UK; UCL Institute of Neurology, London, WC1N 3BG, UK; 1 Department of Brain Sciences, Imperial College London, London, W12 0BZ, UK; Department of Neuroimaging, Institute of Psychiatry, Psychology and Neuroscience, King's College London, London, SE5 8AF, UK; 1 Department of Brain Sciences, Imperial College London, London, W12 0BZ, UK; Dementia Research Institute Care, Research and Technology Centre, Imperial College London, London, W12 0BZ, UK; 1 Department of Brain Sciences, Imperial College London, London, W12 0BZ, UK; Dementia Research Institute Care, Research and Technology Centre, Imperial College London, London, W12 0BZ, UK; UCL Institute of Neurology, London, WC1N 3BG, UK; UK Dementia Research Institute at UCL, London, WC1N 3BG, UK; UKDRI Fluid Biomarker Laboratory, London, WC1N 3BG, UK; UCL Institute of Neurology, London, WC1N 3BG, UK; UK Dementia Research Institute at UCL, London, WC1N 3BG, UK; UKDRI Fluid Biomarker Laboratory, London, WC1N 3BG, UK; Department of Psychiatry and Neurochemistry, Institute of Neuroscience and Physiology, the Sahlgrenska Academy, University of Gothenburg, Mölndal, 431 41, Sweden; Clinical Neurochemistry Laboratory, Sahlgrenska University Hospital, Mölndal, 413 45, Sweden; Hong Kong Center for Neurodegenerative Diseases, Hong Kong, China; Rugby Football Union, Twickenham Stadium, Twickenham, Middlesex TW2 7BA, UK; London School of Hygiene and Tropical Medicine, London, WC1E 7HT, UK; UCL Institute of Neurology, London, WC1N 3BG, UK; 1 Department of Brain Sciences, Imperial College London, London, W12 0BZ, UK; Dementia Research Institute Care, Research and Technology Centre, Imperial College London, London, W12 0BZ, UK

**Keywords:** rugby, head injury, cortical thickness, MRI, GFAP

## Abstract

There is growing concern that elite rugby participation may negatively influence brain health, but the underlying mechanisms are unclear. Cortical thickness is a widely applied biomarker of grey matter structure, but there is limited research into how it may be altered in active professional rugby players. Cross-sectional MRI data from 44 active elite rugby players, including 21 assessed within 1 week of head injury, and 47 healthy controls were analysed. We investigated how active elite rugby participation with and without sub-acute traumatic brain injury influenced grey matter structure using whole cortex and region of interest cortical thickness analyses. Relationships between cortical thickness and biomarkers of traumatic brain injury, including fractional anisotropy, plasma neurofilament light and glial fibrillary acidic protein, were also examined. In whole-cortex analyses, precentral cortical thickness in the right hemisphere was lower in rugby players compared with controls, which was due to reductions in non-injured players. *Post hoc* region of interest analyses showed non-injured rugby players had reduced cortical thickness in the inferior precentral sulcal thickness bilaterally (*P* = 0.005) and the left central sulcus (*P* = 0.037) relative to controls. In contrast, players in the sub-acute phase of mild traumatic brain injury had higher inferior precentral sulcal cortical thickness in the right hemisphere (*P* = 0.015). Plasma glial fibrillary acidic protein, a marker of astrocyte activation, was positively associated with right inferior precentral sulcal cortical thickness in injured rugby players (*P* = 0.0012). Elite rugby participation is associated with localized alterations in cortical thickness, specifically in sulcal motor regions. Sub-acute changes after mild traumatic brain injury are associated with evidence of astrocytic activation. The combination of cortical thickness and glial fibrillary acidic protein may be useful in understanding the pathophysiological relationship between sporting head injury and brain health.

## Teaser

There is concern that elite rugby participation adversely influences brain health. Parker *et al*. show that in-game head injury may lead to localized cortical swelling that correlates with a marker of astrocytic activation, whereas the same region exhibit reductions in cortical thickness that may occur in the longer term.

## Graphical Abstract

**Graphical Abstract:**
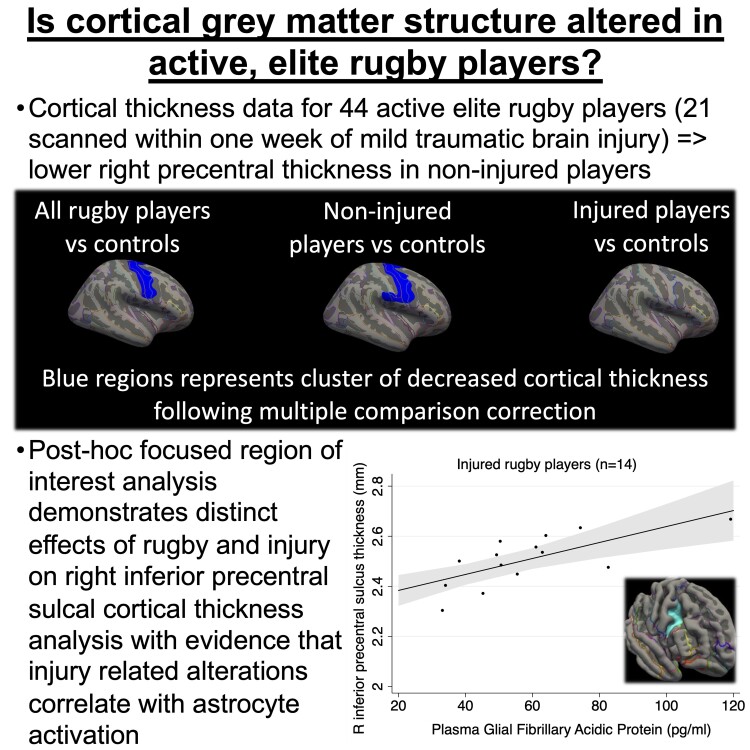

## Introduction

Rugby is a widely played collision team sport with high rates of head injury, and there is significant interest in how elite rugby participation, as well as other elite sports associated with repetitive head injury, influence brain health in the long term.^1-7^ Recent epidemiological data suggest that retired rugby union players are at increased risk of being diagnosed with neurodegenerative conditions.^7^ However, understanding the precise pathophysiological changes that occur during active elite rugby participation and how they may mechanistically increase any risk of future neurodegeneration is limited.

Research utilizing *in vivo* biomarkers relevant to head injury and neurodegeneration during a player’s career is vital to better understand how elite rugby participation influences brain health. Work using blood-based biomarkers in UK-based elite rugby players has shown that concentrations of both plasma neurofilament light (NfL), a marker of axonal injury, and plasma glial fibrillary acidic protein (GFAP), a marker of astrocyte activation, are elevated following in-game head injury associated with symptoms of mild traumatic brain injury (TBI). These blood biomarkers had high diagnostic accuracy for discriminating injured and non-injured players.^2^ Furthermore, diffusion tensor imaging, which has been shown to be a reliable biomarker of traumatic axonal injury in TBI and can be applied clinically at the individual level, has also been utilized to investigate the pathophysiological changes associated with rugby participation.^4,8^ We have previously reported imaging results from 44 active elite players based in the UK, including 21 assessed within 1 week of head injury, and revealed evidence of either white matter diffusion tensor imaging abnormalities or traumatic vascular injury (microbleeds on susceptibility-weighted imaging), in approximately 23% of participants.^4^ These changes were most evident in non-injured players, rather than those who had recently experienced a head injury, suggesting that these effects are most likely related to chronic high-level rugby participation.^4^ Furthermore, the corticospinal tract was the most frequently affected white matter tract suggesting a particular vulnerability of the motor system in the context of elite rugby participation.^4^

Whilst there is emerging evidence relating rugby participation at the elite level to changes in blood-based biomarkers and white matter abnormalities on imaging, the relationship between active elite rugby participation and *in vivo* measurements of cortical grey matter structure is uncertain.^9^ Grey matter atrophy is a key feature of many neurodegenerative diseases, including chronic traumatic encephalopathy (CTE), a tauopathy typically associated with a history of repetitive head impacts, where pathological change is largely concentrated in the cortical sulci of the frontal and temporal lobes.^10-13^ Aligned to this, computational modelling has demonstrated brain tissue deformation is greatest in the cortical sulci following sports-related head injury highlighting a possible mechanistic link between repetitive head impacts and the location of CTE pathology.^14^

MRI-derived estimates of cortical thickness have been shown to be a helpful biomarker of neurodegeneration in several settings such as Alzheimer’s disease and motor neuron disease (MND).^15-18^ Cortical thickness represents an attractive biomarker of cortical grey matter structure in the context of sports-related repetitive head trauma owing to the widely available nature of volumetric T_1_-weighted MRI and the anatomical location of pathologies associated with repetitive head impacts.^10,14,19,20^

Here, we focus on investigating grey matter structure in active elite rugby players, using data from 44 active elite rugby players, including 21 acquired within 1 week of mild (probable) TBI, and 47 healthy controls to investigate the influence of active elite rugby participation on cortical thickness.^4^ The primary hypotheses tested were as follows: (i) elite rugby players will have evidence of altered cortical thickness; (ii) observed differences will be more prominent in sulcal regions; and (iii) cortical thickness will be related to biomarkers of axonal injury and/or neuroinflammation.

## Materials and methods

### Participants

Data from 44 elite rugby players (41 male and 3 female) and 47 healthy controls (34 male and 13 female) were analysed.^4^ Twenty-one players were assessed within 1 week of a head injury that met the criteria for mild TBI using the Mayo Clinic classification (sub-acutely injured rugby players), whilst the remaining 23 players did not report a recent head injury with symptoms suggesting TBI.^21^ No participants had a diagnosis of a major neurological/psychiatric disorder. All controls were screened for a past history of TBI; 15/47 controls were active athletes. Athletic participation was defined as individuals who underwent greater than 6 h of exercise per week. No controls had a history of competitive collision sport participation. All participants provided written informed consent as per the Declaration of Helsinki. Ethical approval was granted by the University College London research ethics committee (7385/001).

### MRI acquisition

T_1_-weighted volumetric MRI data magnetization prepared rapid gadient echo, 1 mm^3^ voxels, 160 slices, field of view (FOV) = 256 mm^2^, repetition time (TR) = 2300 ms, echo time (TE) = 2.98 ms and flip angle = 9°) were acquired on a Siemens 3 T scanner using a 32-channel head coil and processed using FreeSurfer version 6.0 to generate cortical thickness data.^22^ Multi-shell diffusion-weighted MRI data (2 mm^3^ voxels, 66 slices, FOV = 256 mm^2^, TR = 5000 ms, TE = 85 ms, *b*-value1 = 700 s/mm^2^ 30 directions, *b*-value2 = 2000s/mm^2^ 60 directions, 6 direction *b*-value = 0 s/mm^2^ and 6 reverse phase encoding direction *b*-value = 0 s/mm^2^ volumes) were acquired.^4^

### Statistical analysis

#### Cortical thickness analysis

In the first instance, an unbiased vertex-wise analysis comparing cortical thickness in rugby players with age-matched controls (adjusted for sex) was performed in FreeSurfer. Surface-based permutation testing for multiple comparison correction across both hemispheres was conducted with the following parameters: smoothing kernel = 15 mm full width at half maximum, 1000 permutations and a cluster-forming threshold of *P* < 0.05.^23^ Additional analyses comparing non-injured players only to controls, and sub-acutely injured rugby players only to controls, were also performed.

In order to better characterize the whole-cortex findings, regions were selected from the Destrieux sulco-gyral atlas to perform constrained exploratory *post hoc* region of interest (ROI) analyses.^24^ The purpose of this exploratory *post hoc* ROI analysis was (i) to identify whether specific cortical regions within an observed cluster of difference have greater rugby-associated differences than others to test the hypothesis that sulcal regions may show greater rugby-specific effects than gyral regions; (ii) to identify if sub-acute injury had distinct effects on cortical thickness than active elite rugby participation; (ii) to provide a more precise estimation of effects sizes; and (iv) to further explore the laterality of any observed effects.

**Figure 1:**
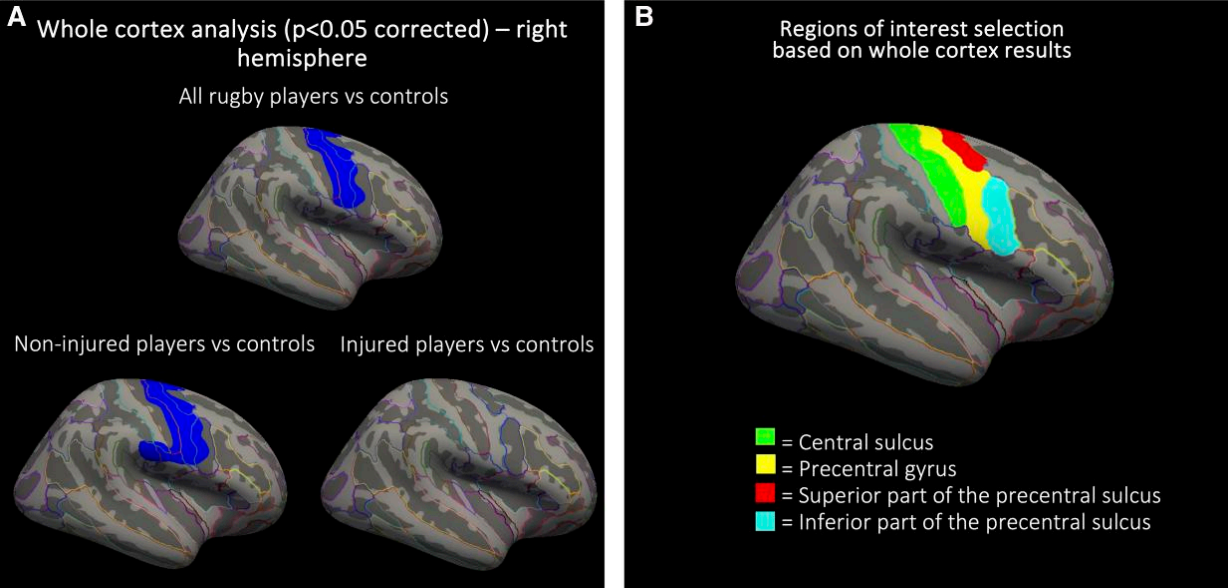
**Cortical thickness analysis in active elite rugby players.** (**A**) Whole-cortex analysis with surface-based permutation testing to correct for multiple comparisons revealing a cluster of lower right hemisphere precentral cortical thickness in all rugby players compared with controls—cluster size = 3718 mm^2^; Montreal Neurological Institute space (MNI) coordinates of vertex of cortical maxima: *x* = 48.8, *y* = −6.5 and *z* = 40.5; cluster-wide *P*-value = 0.037 (cluster-wide *P*-value confidence interval 0.028–0.049), as well as non-injured players only compared with controls—cluster size = 4878 mm^2^; MNIcoordinates of vertex of cortical maxima: *x* = 58.9, *y* = 2.6 and *z* = 27.1; cluster-wide *P*-value = 0.014 (cluster-wide *P*-value confidence interval 0.008–0.019). No statistically significant differences were observed between injured players and controls. (**B**) ROIs selected for *post hoc* linear regression analysis based on whole-cortex results.

Based on the whole-cortex analysis results (Fig. 1), the following ROIs were selected: central sulcus, precentral gyrus, superior part of the precentral sulcus and inferior part of the precentral sulcus.

Linear regression models with robust standard errors were performed across all participants to investigate the distinct effects of active elite rugby participation (i.e. non-injured rugby players compared with controls) and recent mild TBI (non-injured rugby players compared with sub-acutely injured rugby players) on cortical thickness in each ROI. All analyses were adjusted for age, sex and athletic participation. Results were expressed in relation to a standard statistical threshold of *P* < 0.05, as well as a more conservative Bonferroni threshold of *P* < 0.006 (corrected across the eight ROIs examined). Additional sensitivity analyses constrained to male participants only, and with non-athletic controls excluded, were also performed given the uneven sex balance observed between groups and the potential confounding effect of physical fitness on brain structure. An additional sensitivity analysis using mean cortical thickness across the entire cortex as the dependent variable was performed to explore whether alterations observed were more likely to be localized or part of a more global effect.

#### Diffusion tensor imaging analysis

Diffusion-weighted MRI data were processed using tract-based spatial statistics to generate skeletonized fractional anisotropy (FA) maps and mean FA values for specific ROI tracts as a marker of white matter tract microstructural integrity as per previously published work.^4^ In ROIs where associations between rugby and altered cortical thickness were observed, analyses incorporating estimates of corticospinal tract FA were performed to investigate how white matter tract integrity may influence cortical thickness in the context of elite rugby participation. The corticospinal tract was selected as the primary tract of interest on anatomical grounds given its strong connections to the precentral cortex implicated in the initial cortical thickness analysis (please see ‘Results’ section), and previous data demonstrate that the corticospinal tract is a prominent site of diffusion tensor imaging abnormalities in elite rugby players.^4^

#### Blood-based biomarker analysis

A subset of injured rugby players (*n* = 14) included in previous work underwent venepuncture on the same day as scanning.^2^ All athletic controls (*n* = 15) also underwent venepuncture on the same day as scanning. Seven participants with sub-acute injury did not undergo blood-based biomarkers on the day of scanning. Same day as scanning, samples were not available for non-injured players or non-athletic controls. Blood samples were centrifuged within 20–60 min with supernatants initially stored at −20°C and then transferred to −80°C within 2 weeks. Plasma NfL and GFAP concentrations were measured using a Quanterix Simoa 4-Plex assay.^2^

In ROIs where there was evidence of associations between sub-acute injury and cortical thickness, additional linear regression models incorporating plasma NfL or GFAP were performed to test the hypothesis that injury-related alterations in cortical thickness were associated with blood-based biomarkers of axonal injury and neuroinflammation.

## Results

Demographics are summarized in Table 1. Rugby players and controls were well matched for age, but there was a higher proportion of males in the rugby group.

**Table 1:** Cortical thickness analysis in active elite rugby players: demographics

Demographics	Non-acutely injured rugby players (*n* = 23)	Sub-acutely injured rugby players (*n* = 21)	Non-rugby controls (*n* = 47, includes 15 athletic controls)
Age, years, mean ± SD	25.4 ± 3.3	25.0 ± 3.8	24.3 ± 3.9
Male sex, *n* (%)	20 (87%)	21 (100%)	34 (72%)^a^
Career length, years, mean ± SD	6.0 ± 3.3	5.3 ± 3.8	n/a
Self-report previous concussions, *n*, mean ± SD	2.3 ± 2.7	3.0 ± 3.3	n/a
Time since injury, days, mean ± SD	n/a	4.7 ± 1.2	n/a
Return to play duration, days, mean ± SD	n/a	7.2 ± 1.5	n/a
Right-handed, *n* (%)	26 (93%)	15 (94%)	data not available

^a^Two-sample Wilcoxon rank-sum (Mann–Whitney) test, all rugby players versus controls, *P* < 0.05.

### Rugby players have decreased right precentral cortical thickness compared with controls at the whole-cortex level

Whole-cortex analysis revealed a cluster of reduced cortical thickness in the right hemisphere precentral region in rugby players compared with controls following permutation-based correction for multiple comparisons (cluster size = 3718 mm^2^; Montreal Neurological Institute space (MNI) coordinates of vertex of cortical maxima: *x* = 48.8, *y* = −6,5 and *z* = 40.5; cluster-wide *P*-value = 0.037). Similar results were evident when comparing non-injured players only to controls (cluster size = 4878 mm^2^; MNI coordinates of vertex of cortical maxima: *x* = 58.9, *y* = 2.6 and *z* = 27.1; cluster-wide *P*-value = 0.014), whilst there was no evidence of difference between injured players and controls (Fig. 1).

### Distinct effects of rugby and sub-acute injury on cortical thickness

**Figure 2:**
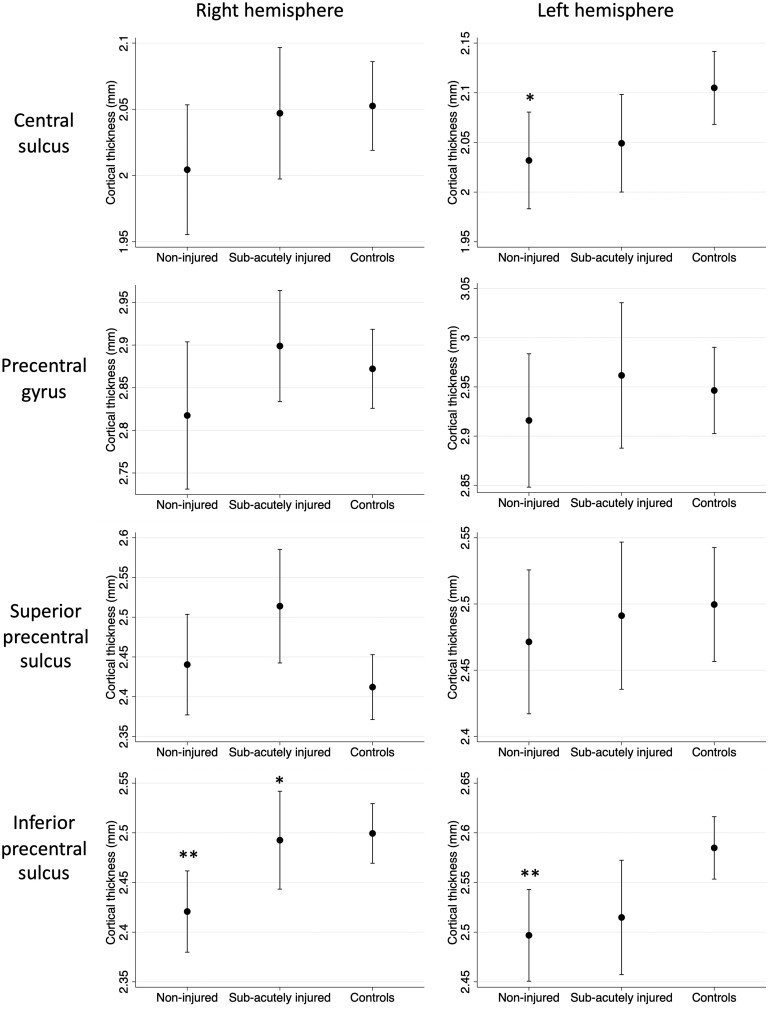
***Post hoc* ROI analysis.** Marginal mean cortical thickness in non-injured rugby players, sub-acute injured rugby players and controls with 95% confidence interval following adjustment for age, sex and athletic participation. *P < 0.05; **P < 0.006 (significant differences highlighted above non-injured rugby players are relative to controls, whilst significant differences highlighted above sub-acutely injured players are relative to non-injured players).

The discrepancy in whole-cortex results between analyses comparing non-injured players to controls and injured players to controls suggested distinct effects of rugby participation and sub-acute head injury. To further investigate the alterations observed within the precentral region further, *post hoc* ROI linear regression analyses were performed to assess the independent effects of rugby and sub-acute injury on cortical thickness across all participants after correction for age, sex and athletic participation. We specifically looked at ROIs from the Destrieux sulco-gyral atlas that fell within the cluster of difference identified during the whole-cortex analysis (Table 2 and Fig. 2). This also enabled the identification of whether specific cortical regions within an observed cluster of difference have greater rugby-associated differences than others to test the hypothesis that sulcal regions may show greater rugby specific effects than gyral regions.

**Table 2:** Mean differences in cortical thickness (mm) between groups following linear regression modelling with robust standard errors adjusted for age, sex and athletic participation

ROI	Mean cortical thickness across all participants		Mean difference between non-injured rugby players and controls		Mean difference between injured and non-injured players	
	Left	Right	Left	Right	Left	Right
Central sulcus	2.07 mm (SD = 0.11)	2.04 mm (SD = 0.10)	−0.073 mm (*P* = 0.037*)	−0.048 mm (*P* = 0.15)	+0.017 mm (*P* = 0.59)	+0.043 mm (*P* = 0.20)
Precentral gyrus	2.94 mm (SD = 0.13)	2.86 mm (SD = 0.17)	−0.031 mm (*P* = 0.51)	−0.066 mm (*P* = 0.29)	+0.046 mm (*P* = 0.31)	+0.08 mm (*P* = 0.11)
Precental sulcus—superior	2.49 mm (SD = 0.11)	2.44 mm (SD = 0.14)	−0.028 mm (*P* = 0.48)	−0.028 mm (*P* = 0.5)	+0.02 mm (*P* = 0.59)	+0.074 mm (*P* = 0.097)
Precentral sulcus—inferior	2.55 mm (SD = 0.11)	2.48 mm (SD = 0.11)	−0.088 mm (*P* = 0.005**)	−0.079 (*P* = 0.005**)	+0.018 mm (*P* = 0.6)	+0.072 mm (*P* = 0.015*)

SD, standard deviation. *P < 0.05; **P < 0.006.

Non-injured players had reduced cortical thickness relative to controls in the inferior precentral sulcus bilaterally relative to controls independent of age, sex and athletic participation, with a 0.088 mm reduction in left inferior precentral sulcus thickness (95% confidence interval 0.027–0.149 mm; *P* = 0.005) and a 0.079 mm reduction in right inferior precentral sulcus thickness (95% confidence interval 0.024–0.136 mm; *P* = 0.005). Non-injured players also had reduced cortical thickness relative to controls in the left central sulcus (0.073 mm lower, 95% confidence interval 0.004–0.141 mm; *P* = 0.037). All other regions examined were non-significant but were directionally consistent (i.e. with a trend for rugby to be associated with reduced cortical thickness).

In contrast, sub-acutely injured rugby players exhibited a 0.072 mm increase in right inferior precentral sulcus cortical thickness relative to non-injured players (95% confidence interval 0.014–0.129 mm; *P* = 0.015). All other regions examined were non-significant.

Additional sensitivity analyses constrained to male participants only, and with non-athletic controls excluded (*n* = 56), were also performed given the uneven sex balance observed between groups and the potential confounding effect of physical fitness on brain structure. Except for the difference between non-injured players and controls in the left central sulcus, where only a trend association was observed in the smaller sample (*P* = 0.078), this approach did not result in any material change in the nature of the associations observed (Table 3). An additional sensitivity analysis using mean cortical thickness across the entire cortex was performed in order to explore whether alterations observed were more likely to be localized or part of a more global effect. There was a non-significant difference of 0.01 mm in mean global cortical thickness between non-injured players relative to controls (95% confidence interval −0.088 to 0.067 mm; *P* = 0.79) following correction for age, sex and athletic participation. Sub-acutely injured rugby players exhibited a trend of 0.062 mm increase in mean global cortical thickness cortical thickness relative to non-injured players, although this was non-significant (95% confidence interval −0.007 to 0.13 mm; *P* = 0.078).

**Table 3:** Sensitivity analysis

ROI	Mean cortical thickness across all participants		Mean difference between non-injured rugby players and controls		Mean difference between injured and non-injured players	
	Left	Right	Left	Right	Left	Right
Central sulcus	2.08 mm (SD = 0.12)	2.05 mm (SD = 0.1)	−0.064 mm (*P* = 0.078)	−0.041 mm (*P* = 0.24)	+0.011 mm (*P* = 0.72)	+0.35 mm (*P* = 0.29)
Precentral gyrus	2.96 mm (SD = 0.14)	2.88 mm (SD = 0.13)	−0.028 mm (*P* = 0.56)	−0.024 mm (*P* = 0.61)	+0.046 mm (*P* = 0.33)	+0.037 mm (*P* = 0.4)
Precental sulcus—superior	2.49 mm (SD = 0.11)	2.46 mm (SD = 0.13)	−0.025 mm (*P* = 0.55)	−0.044 mm (*P* = 0.29)	+0.016 mm (*P* = 0.67)	+0.052 mm (*P* = 0.22)
Precentral sulcus—inferior	2.56 mm (SD = 0.11)	2.49 mm (SD = 0.10)	−0.093 mm (*P* = 0.004**)	−0.080 (*P* = 0.006**)	+0.025 mm (*P* = 0.48)	+0.076 mm (*P* = 0.013*)

Mean differences in cortical thickness between groups following linear regression modelling with robust standard errors adjusted for age using data with females and non-athletic controls excluded (*n* = 56). ROI, region of interest; SD, standard deviation. *P < 0.05; **P < 0.006.

### Biomarkers of TBI and their relationship with cortical thickness

Previous work has revealed the corticospinal tract to be a prominent site of impaired white matter tract integrity in active elite rugby players from this cohort.^4^ However, there was no evidence that corticospinal tract white matter integrity predicted cortical thickness in regions where associations between rugby and altered cortical thickness were observed. Specifically, there was no association between right corticospinal tract FA and right inferior precentral sulcus cortical thickness in non-injured players (*β* = −0.82; 95% confidence interval −2.44 to 0.81; *P* = 0.31) or in sub-acutely injured players (*β* = 1.68; 95% confidence interval −1.22 to 0.02; *P* = 0.24). In addition, there was no evidence that left corticospinal tract FA predicted cortical thickness in the left inferior precentral sulcus (*β* = −0.59; 95% confidence interval −2.73 to 1.55; *P* = 0.57) or the left central sulcus (*β* = 0.26; 95% confidence interval −1.77 to 2.30; *P* = 0.79) in non-injured rugby players.

**Figure 3:**
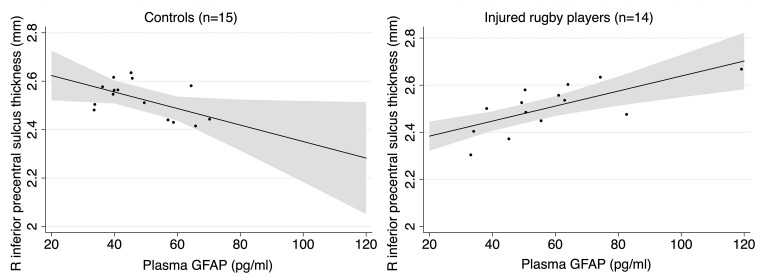
**GFAP and cortical thickness.** Distinct associations between thickness of the inferior part of the right precentral sulcus and plasma GFAP with 95% confidence intervals following adjustment for age in sporting controls with no history of elite rugby participation/head injury (left, *β* = −0.0035; 95% confidence interval −0.0067 to −0.001; *P* = 0.041) and sub-acutely injured rugby players (right, *β* = +0.0032; 95% confidence interval +0.0015 to +0.0048; *P* = 0.0012) with blood sampling on day of scan. Dots represent individual thickness values.

We next examined whether right inferior precentral sulcus cortical thickness was correlated with blood-based biomarkers of neuroinflammation or axonal injury measured with GFAP and NfL (this was restricted to sub-acutely injured players due to data availability). Plasma GFAP positively correlated with right inferior precentral cortical thickness in sub-acutely injured rugby players (*β* = +0.0032; 95% confidence interval +0.0015 to +0.0048; *P* = 0.0012—see Table 4 and Fig. 3). In contrast to the injured players, plasma GFAP was negatively correlated with inferior precentral cortical thickness in athletic controls (*β* = −0.0035; 95% confidence interval −0.0067 to −0.001; *P* = 0.041—see Table 4 and Fig. 3). This interaction between GFAP and sub-acute injury was not observed in any other regions examined. There were no associations observed between plasma NfL and right inferior precentral cortical thickness.

**Table 4:** The relationship between effects of injury on right inferior precentral cortical thickness and blood-based biomarkers

	Sub-acutely injured rugby players (*n* = 14)	Controls (*n* = 15)
Mean GFAP pg/mL (SD)	58.59 (22.6)	48.03 (2.23)
GFAP *β*-coefficient for right inferior precentral sulcus cortical thickness (*P*-value)	0.0032 (*P* = 0.0012**)	−0.0035 (*P* = 0.041*)
Mean NfL pg/mL (SD)	10.29 (6.06)	4.95 (2.23)
NfL *β*-coefficient for right inferior precentral sulcus cortical thickness (*P*-value)	−0.0013 (*P* = 0.65)	−0.0035 (*P* = 0.71)

All analyses were additionally corrected for age and sex. GFAP, glial fibrillary acidic protein; NfL, neurofilament light; SD, standard deviation. *P < 0.05; **P < 0.06.

## Discussion

We demonstrate that active elite rugby participation is associated with localized reductions in cortical grey matter thickness, with the strongest evidence in the inferior part of the precentral sulcus. In contrast, players in the sub-acute phase of mild TBI had evidence of *higher* inferior precentral sulcal cortical thickness in the right hemisphere, and plasma GFAP, a marker of astrocyte activation, was positively associated with right inferior precentral sulcal cortical thickness in injured rugby players. These distinct relationships between active elite rugby participation and sub-acute injury may give potential insights into the pathophysiological relationships between head injury in elite rugby and brain health.

Given its cross-sectional nature and small sample size, it is important to exercise caution when inferring the causal direction of the associations observed. However, the decreases in cortical thickness observed in non-acutely injured rugby players raise the question of whether this may represent early neurodegenerative change. Cortical grey matter atrophy is a key feature of many neurodegenerative diseases, and cortical thickness has been shown to be a useful biomarker in a neurodegenerative context.^11,15-17,25^ The anatomical pattern of our findings may be related to the pattern of underlying brain injury and associated neurodegeneration. For example, CTE is typified by tau deposition and atrophy in the depths of the cortical sulci and has been described in former rugby players at post-mortem.^11,26-29^ Furthermore, computational modelling has demonstrated head impact–induced brain tissue deformation is often greatest in cortical sulci.^14,19^ Our observation that changes in cortical thickness are mainly seen in sulcal regions is in keeping with early changes of neurodegeneration being seen in areas exposed to high strain rates and subsequent injury as a result of repetitive head impacts. However, it is important to note that CTE is currently a post-mortem diagnosis and specific *in vivo* biomarkers are lacking.^30^ Furthermore, the motor regions identified as being potentially altered in this study have not been specifically reported to be involved at an early stage in CTE on post-mortem studies highlighting this may represent a distinct process to CTE,^11,26-29^ although it is important to note that large-scale post-mortem studies specifically in rugby players are lacking and establishing whether different sports have different anatomical patterns of CTE pathology in the future will be of interest.

In terms of previous similar studies, one previously published study did not find evidence of cortical thickness differences between rugby players and controls.^9^ However, the overall sample size was smaller than the work presented in this paper and included both active and retired players over a wider age range. Furthermore, data from other sports with a high incidence of head injuries, such as American football, have also shown associations between participation and lower cortical thickness.^20^

We also investigated a subgroup of players shortly after head injuries that occurred in rugby matches. In contrast to the decreased cortical thickness observed between non-injured rugby players and controls, sub-acute injury in rugby players was associated with increased sulcal thickness relative to non-injured players. Furthermore, increased sulcal thickness in the precentral sulcus was associated with increased plasma GFAP in injured rugby players. Given the cross-sectional nature and small sample size, it is difficult to draw any firm conclusions about causation. However, one interpretation of these results is that local sulcal injury produced by high strains at the time of head impact may result in astrocytic activation (indexed by plasma GFAP) and subsequent local swelling of the cortex produced by neuroinflammation associated with injury. Plasma GFAP is known to be elevated following in-game head injury in rugby players.^2^ This may be relevant to the triggering of progressive neurodegeneration after head injury as astrocyte activation and neuroinflammation are key mediators of neurodegeneration in a range of diseases.^31^ Exploring the role of neuroinflammation and its downstream effects on cortical grey matter structure will be an important focus of future work.

Our findings were localized to a motor cortical region, which is intriguing given recent evidence that rugby participation may be associated with increased MND risk.^7^ Involvement of the motor cortex also suggests that these changes are likely to be distinct from typical Alzheimer’s disease, where the motor cortex is relatively spared from cortical grey matter atrophy.^15,17,18^ We have previously reported corticospinal tract abnormalities in a high proportion of elite rugby players, measured using diffusion imaging in the same cohort.^4^ Furthermore, exercise and participation in sports associated with repetitive head injury have been implicated as risk factors for MND.^6,32,33^ Taken together, although speculative, the findings may suggest that the motor system may be particularly vulnerable to the adverse effects of repeated head injuries. Given the high-intensity nature of elite rugby, it is possible that the increased metabolic demands placed on the motor system may increase its vulnerability to trauma-associated damage, with excitotoxicity, oxidative stress and neuroinflammation possible mechanisms.^34^ Although there is evidence that the relative risk for MND may be increased in rugby players, the absolute risk is still low.^7^ The pathogenesis of MND has been conceptualized as a multi-step process, and it is extremely unlikely that trauma-related abnormalities in the motor system structure would be the sole reason for MND development. This is likely to be a multi-factorial process, with other things important, particularly genetics. Understanding interactions between environmental and genetic risk factors for neurodegenerative diseases is an important area of future research.^35^ It is important to note that it is possible that there are more global effects beyond the motor cortex, although we did not find strong evidence of this when looking at mean cortical thickness across the entire thickness in sensitivity analyses. Again, larger-scale work will be required to explore this further.

Given the relatively localized nature and the relatively small effect size, the more immediate functional consequences of these findings may be limited, which is supported by the fact that all participants were still engaged in rugby at the elite level. However, the long-term implications of these findings for brain health in elite rugby players are unknown. The findings highlight the need for a large-scale longitudinal study of elite rugby players, with a focus on clinical outcomes, detailed biomarker characterization and ultimately neuropathological examination.

This study is limited by its small sample size, which reduces statistical power to identify differences in cortical thickness, particularly at the whole-cortex level, where strict multiple comparisons correction is vital to limit false-positive results.^23^ However, the effect of rugby on the inferior precentral cortex thickness and the relationship between right hemisphere inferior precentral cortex thickness and plasma GFAP in injured rugby players showed strong effects that survived Bonferroni-based correction over the eight regions examined (*P* < 0.006). Previous work has shown significant heterogeneity across the cortical surface in terms of the sample sizes required to show significant group differences in cortical thickness.^36^ Future studies with larger sample sizes may be required to identify potentially rugby-related differences in cortical thickness in other brain regions.

A further limitation of this study is that many of the participants did not have blood-based biomarker quantification on the same day of scanning, which meant we were only able to investigate the relationships between plasma NfL and GFAP and cortical thickness in the context of sub-acutely injured rugby players. Future work looking at the relationship between blood-based biomarkers such as plasma NfL and GFAP with cortical thickness in non-acutely injured players will be of significant interest. The selective nature of this cohort is also a limitation. In particular, there was limited data available for female athletes. Larger-scale prospective studies enrolling a broader range of players of both sexes at an earlier stage of their career with longitudinal follow-up would help address such bias. A further limitation of the study is the lack of detailed characterization of the number of repetitive head impacts each player experienced throughout their career. There is mounting evidence that these often asymptomatic head impacts have distinct biological effects to mild TBI.

Although career length may be a useful proxy marker of exposure in retired athletes, the substantial collinearity between age and career length limits its usefulness in active players. Further work incorporating more detailed quantification of repetitive head impacts, using mouthguard accelerometers for example or factoring in factors such as player position, will be helpful in addressing this issue in future work.^37^

In conclusion, we show that rugby participation at the elite level is associated with localized alterations in cortical thickness, specifically in sulcal motor regions. Sub-acute changes after mild TBI are associated with evidence of astrocytic activation. The combination of cortical thickness and GFAP may be useful in understanding the pathophysiological relationship between sporting head injury and brain health. Furthermore, the anatomical location of our findings does hint at potential mechanistic links between elite rugby participation and specific neurodegenerative pathologies, such as CTE and MND, and should be the focus of future study.

## Data Availability

The data that support the findings of this study are available from the corresponding author, upon reasonable request.
